# Phylogenetic Network Models as Graphical Models

**DOI:** 10.1007/s11538-026-01695-3

**Published:** 2026-07-13

**Authors:** Seth Sullivant

**Affiliations:** Department of Mathematics, North Carolina State University, Raleigh, NC 27695 USA

**Keywords:** Phylogenetic network models, graphical models, identifiability

## Abstract

The displayed tree phylogenetic network model is shown to sit as a natural submodel of the graphical model associated to a directed acyclic graph (DAG). This representation allows us to derive a number of results about the displayed tree model. In particular, the concept of a local modification to a DAG model is developed and applied to the displayed tree model. As an application, some nonidentifiability issues related to the displayed tree models are highlighted as they relate to reticulation edges and stacked reticulations in the networks. We also derive rank conditions on flattenings of probability tensors for the displayed tree model, generalizing classic results for phylogenetic tree models.

## Introduction

Phylogenetic trees are the basic object used to represent the evolutionary relationships between a collection of taxa (Felsenstein 2003; Steel 2016). However, there are many situations when a more complex network structure is necessary to describe the history of a collection of species. The network structure can take into account hybridization and more general types of reticulate evolution.

Once a particular network is specified, there are numerous models for how evolution might occur on that network. The network multispecies coalescent is a widely used model that allows for both the population level effect of incomplete lineage sorting, and for hybridization (Yan 2012). It is usually used as a process for generating gene trees, with a separate substitution process of the evolution of sequences along those gene trees. Questions about identifiability in the network multispecies coalescent have often involved inferring the species network from gene trees, and there are many different approaches to such identifiability results (Allman et al. 2024, 2023).

An alternate family of network models consider evolution of sequences on the tree contained within that network, but without the coalescent process. This model is sometimes called the *displayed tree model*, because the only gene trees that can be created are the trees that are displayed by the network. Identifiability questions around the displayed trees model have been addressed in a few papers (Gross and Long 2018; Gross et al. 2021). Those works specifically address the level-1 networks for group-based models. Note that the displayed tree model can be thought of as a limiting family of the network multispecies coalescent, where all coalescent events occur at the same time as speciation (John 2025).

In this note we explain how to think of the displayed tree phylogenetic network model as a natural submodel of a corresponding graphical model on a directed acyclic graph (DAG). DAG graphical models are a commonly used family of statistical models that give a recursive factorization of a joint probability distribution based on the underlying graph structure. This representation of the displayed tree model as a DAG model is useful for proving some results about network models by using properties of DAG models, like conditional independence and the local structure of the conditional distributions in the model. One feature we highlight here is that the DAG structure often allows us to prove uniform results across all model types (not just group-based models, or the general Markov model).

**Fig. 1:**
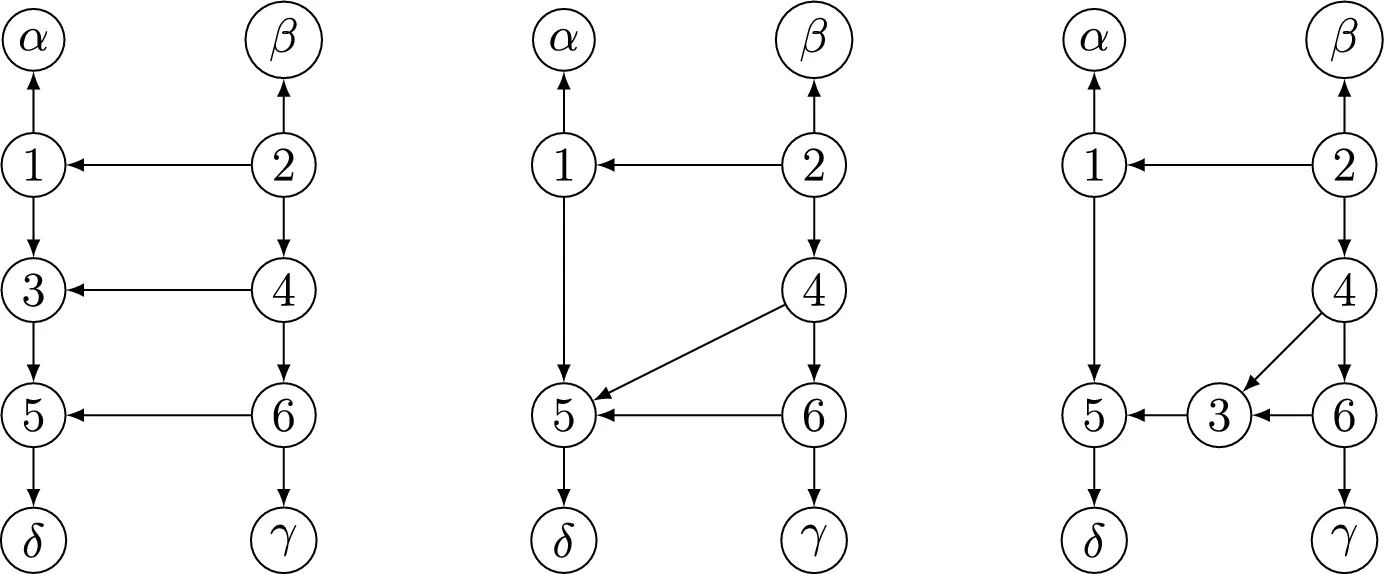
Two binary networks (left and right) and one non-binary network (middle) that give the same distributions on the observed leaves $\alpha , \beta , \gamma , \delta$

One goal for this note is to make the connection between the displayed tree model and the DAG models more widely known. We use this fact to prove some straightforward results about the displayed tree model, and we want to advertise this perspective in the hope that might be useful for proving other results about phylogenetic network models. Along the way, we see some surprising nonidentifiability results for the displayed tree model, especially as it involves reticulation vertices.

### Example 1.1

As an example of results produced in this note, consider the three networks given in Figure 1. For evolutionary models satisfying common mathematical assumptions (in particular, for the equivariant phylogenetic models), all three networks produce the same family of distributions on sequences at the leaves ($\alpha , \beta , \gamma , \delta$). The sequences of reticulations are not the same in the left and right networks. The issue that causes this phenomenon is the presence of stacked reticulations in the network. Specifically, vertices 3 and 5 are reticulations, and there is an edge from 3 to 5.

The outline of the paper is as follows. Sections 2 and 3 provide background on graphical models and the displayed tree phylogenetic network model and shows how the displayed tree model arises as a submodel of directed acyclic graph models (DAGs). Section 4 introduces the notion of a local modification in a DAG, as a tool to show that two DAGs give the same family of probability distributions. Section 5 explores the consequences of the local modification machinery for the case of stacked reticulations. This produces some situations where two network models produce the same families of probability distributions in rather non-obvious ways. In Sect. 6, some observations are made about loss of identifiability in the conditional distributions associated to reticulation vertices. Finally, in Sect. 7 conditional independence in DAGs is used to derive some results about the ranks of flattenings of probability tensors in the case of the displayed tree model.

## Background on Graphical Models

This section reviews background on directed graphical models that will be useful for studying the displayed tree model. More details can be found in Lauritzen (1996).

Let G=(V,E) be a directed acyclic graph with vertex set *V*. The graph is directed acyclic, in that there are no directed cycles in the graph, though there can be cycles that are not directed. For each i∈V, let $\textrm{pa}(i) = \{j \in V: j \rightarrow i \in E \}$ be the set of parents of a node *i*.

For each i∈V, we have a random variable $X_i$. Let $X = (X_i: i \in V)$, be the random vector. For a set A⊆V, let $X_A = (X_a: a \in A)$ be the subvector with indices indexed by *A*. We assume that all of the random variables are discrete so we can talk about probability distributions (but for continuous random variables, we can use density functions instead). So each random variable $X_i$ has a state space $[r_i]:= \{1,2, \ldots , r_i\}$. We let $\mathcal {R}= \prod _{i \in V} [r_i]$, be the state space of the random vector. For each $x \in \mathcal {R}$, and A⊆V, we can also take subvectors $x_A$. Let $\mathcal {R}_A$ denote the state space of the random vector $X_A$, that is, $\mathcal {R}_A = \prod _{i \in A} [r_i]$.

Define p(x)=P(X=x) to be the joint probability distribution of *X*. In the event that V=[n], we can write this as

$$ p(x) = p(x_1, \ldots , x_n) = P(X_1 = x_1, \ldots , X_n = x_n). $$

For any A⊆V we can compute the marginal distribution

$$ p_A(x_A) = P(X_A = x_A) = \sum _{y_B \in \mathcal {R}_B} p(x_A, y_B). $$

For A,B⊆V, disjoint, we can compute the conditional distribution

$$ p_{A|B}(x_A|x_B) = P(X_A = x_A | X_B = x_B) = \frac{p_{A \cup B}(x_A, x_B)}{p_B(x_B)}. $$

In the special case that $A = \{i\}$ is a singleton, we just use $p_{i|B}(x_i | x_B)$.

Consider the following factorization of the joint probability according to the DAG *G*

1 $$\begin{aligned} p(x) = \prod _{i \in V}p_{i|\textrm{pa}(i)}(x_i | x_{\textrm{pa}(i)}). \end{aligned}$$

Note that not every probability distribution can satisfy this equation. The graphical model associated to the DAG *G* consists of all probability distributions on $\mathcal {R}$ that satisfy (1).

Alternatively, we can also think about (1) as a parametrization of a model, with each of the conditional distributions $p_{i|\textrm{pa}(i)}(x_i | x_{\textrm{pa}(i)})$ as a set of free parameters. Here we work with the constraint that they should actually be conditional probability distributions, that is

1. $$p_{i|\textrm{pa}(i)}(x_i | x_{\textrm{pa}(i)}) \ge 0$$ and
2. $$\sum _{x_i \in [r_i]} p_{i|\textrm{pa}(i)}(x_i | x_{\textrm{pa}(i)} ) = 1$$.

### Example 2.1

**Fig. 2:**
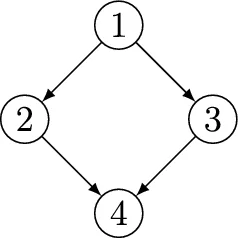
A directed four-cycle $C_4$

Consider the directed four-cycle graph $C_4$ in Figure 2. The parent sets of each of the vertices are $\textrm{pa}(1) = \emptyset$, $\textrm{pa}(2) = \{1\}$, $\textrm{pa}(3) = \{1\}$, $\textrm{pa}(4) = \{2,3\}$. The factorization of the joint distribution induced by $C_4$ is:

2 $$\begin{aligned} p(x_1, x_2, x_3, x_4) = p_1(x_1) p_{2|1}(x_2|x_1) p_{3|1}(x_3|x_1) p_{4|2,3}(x_4 | x_2, x_3). \end{aligned}$$

The graphical model associated to $C_4$ consists of all probability distributions that satisfy the factorization (2).

## The Displayed Tree Model

Phylogenetic network models arise as special cases of the general DAG graphical model by putting multiple types of restrictions on the DAGs that can arise, the particular structure of the conditional distributions that are used, and the fact that many of the variables are unobserved random variables (i.e. hidden random variables or latent random variables). Throughout, all the random variables $X_i$ are assumed to have the same state space Σ (which for many models is $\Sigma = \{\texttt{A},\texttt{C},\texttt{G},\texttt{T}\}$).

First, we describe the restrictions on the DAGs that can arise. Different types of vertices in a network model have different names:

- Root vertices: indegree 0
- Leaf vertices: outdegree 0
- Tree vertices: indegree 1
- Reticulation vertices: indegree >1

Typically (though not exclusively) we assume that there is a single root in *G*. This node represents a common ancestor of all the nodes in the graph. Sometimes the graphs that can arise in network models are further restricted in various ways, but technically any other type of directed acyclic graph could occur.

Often one restricts to binary phylogenetic networks, in which case we assume that the root has degree 2, leaves have degree 1, and all other vertices have degree 3. However, we will see that even if we only care about binary phylogenetic networks, it is useful to look at phylogenetic networks more broadly. There are many other families of network restrictions that one could add which concern the relations between types of vertices in the network. See (Kong et al. 2022) for an extensive survey on different types of phylogenetic networks.

In many modeling contexts with phylogenetic networks, it is useful to consider networks with parallel edges. However, for the displayed tree model with a mutation process that is closed under convex combinations, this model is equivalent to a model without parallel edges, so we can safely ignore this condition and assume our DAGs are simple. This will be discussed in detail in Proposition 5.4. For this note, we do not need to make any other restrictions.

### Example 3.1

**Fig. 3:**
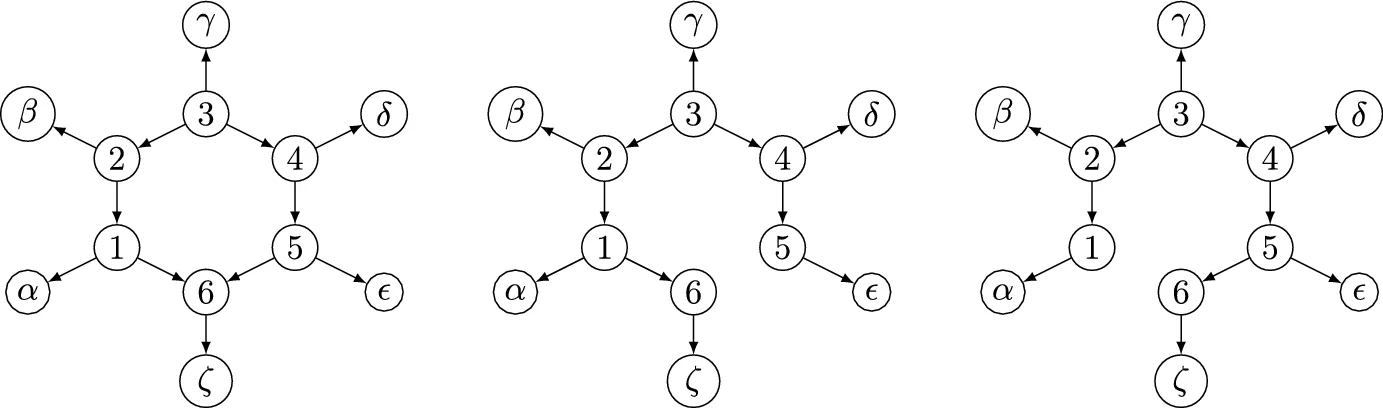
A 6 sunlet network, and its two displayed trees

Consider the network on the left in Figure 3. The root is vertex 3. Vertices $\alpha , \beta , \gamma , \delta , \epsilon ,$ and ζ are leaves. Vertex 6 is a reticulation vertex. All other vertices are tree vertices.

A second consideration when describing a network model, is identifying a set of variables that are the observed variables. In many network modeling scenarios, the leaves of the network are assumed to be the only observed variables, though this assumption is not technically necessary. However, we will assume that each observed node has no descendant node that is hidden. This satisfies the natural assumption that observed variables should correspond to extant taxa, i.e. taxa that we can directly observe. For a given set of observed taxa *O* and hidden taxa *H*, the distribution of states at the observed variables is obtained by marginalizing over *H*, that is

$$ p_O(x_O) = \sum _{x_H \in \mathcal {R}_H} p(x_O, x_H). $$

Finally, we come to the model description which amounts to restrictions on the structure of the conditional distributions $p_{i|\textrm{pa}(i)}(x_i | x_{\textrm{pa}(i)} )$. The restriction in the network displayed tree model amounts to the following restrictions.

- To each edge e=i→j in the network we associate a Markov transition matrix, which is the conditional distribution of $X_j | X_i$. This is denoted $M^{ij}$, with entries $M^{ij}(x_j|x_i)$.
- The matrices $M^{ij}$ are usually not allowed to be arbitrary conditional distributions (as they would be in the graphical model), but rather are restricted to have a particular structure. Example structures include models like the Jukes-Cantor model, general Markov model, Kimura models, HKY model, general time reversible, etc. Matrices might also be required to be of the form exp(Qt) for some rate matrix *Q* and branch length parameter *t*.
- For each reticulation vertex *j* (that is $\# \textrm{pa}(j) > 1$), there is a probability vector $\pi ^j \in \Delta ^{\textrm{pa}(j)}$. The coordinate $\pi ^j_i$ is the probability that the edge i→j is chosen at the reticulation vertex. Note if the non-root vertex *j* is not a reticulation vertex, so it has only one parent *i*, then we can define $\pi ^i_j = 1$. This will be useful in some proofs.
- With these features, we get the following formula for the conditional probability at each reticulation vertex *j*:
  $$ p_{j|\textrm{pa}(j)}(x_j | x_{\textrm{pa}(j)}) = \sum _{i \in \textrm{pa}(j)} \pi ^j_i M^{ij}(x_j|x_i). $$

The displayed tree model is usually presented as follows: for each reticulation vertex *j*, one of the edges i→j is chosen with probability $\pi ^j_i$. The resulting DAG obtained after making all these choices has no reticulation vertices, and hence no cycles, so it is a forest (and with appropriate assumptions on the network, it will be a rooted tree). The probabilities are then calculated according to the graphical model on that forest as described above. This clearly yields the same description of the model as above using the graphical model formulation. Indeed, this can be seen by using the graphical model formulation, and using the distributive law to expand and collect monomials in powers of the $\pi ^j_i$ terms. Note that it suffices to see that the models are the same when all variables are observed variables (since then this will also be true when some of the variables are hidden).

### Example 3.2

Consider the four-cycle graph from Figure 2. There is only one reticulation vertex which is vertex 4. The parametrization for the graphical model in this graph is

$$ p(x_1, x_2, x_3, x_4) = p_1(x_1) p_{2|1}(x_2|x_1) p_{3|1}(x_3|x_1) p_{4|2,3}(x_4 | x_2, x_3). $$

In the displayed tree model, we parametrize $p_{4|2,3}(x_4 | x_2, x_3)$ as

$$ p_{4|2,3}(x_4 | x_2, x_3) = \pi ^4_2 M^{24}(x_4|x_2) + \pi ^4_3 M^{34}(x_4|x_3) $$

and

$$ p_{2|1}(x_2|x_1) = M^{12}(x_2|x_1) \quad p_{3|1}(x_3|x_1) = M^{13}(x_3|x_1) $$

so we get

$$\begin{aligned} p(x_1, x_2, x_3, x_4)= &  \quad p_1(x_1) p_{2|1}(x_2|x_1) p_{3|1}(x_3|x_1) p_{4|2,3}(x_4 | x_2, x_3) \\= &  \quad p_1(x_1) M^{12}(x_2|x_1) M^{13}(x_3|x_1) (\pi ^4_2 M^{24}(x_4|x_2) + \pi ^4_3 M^{34}(x_4|x_3) ) \\= &  \quad \pi ^4_2 p_1(x_1) M^{12}(x_2|x_1) M^{13}(x_3|x_1) M^{24}(x_4|x_2) \\ &  + \pi ^4_3 p_1(x_1) M^{12}(x_2|x_1) M^{13}(x_3|x_1) M^{34}(x_4|x_3). \end{aligned}$$

The first term in this final sum is $\pi ^4_2$ times the probability distribution in the tree obtained by deleting the edge 3→4, and the second term is $\pi ^4_3$ times the probability distribution in the tree obtained by deleting the edge 2→4.

### Example 3.3

For a more phylogenetics relevant example, consider the network in Figure 3 on the left. This has just one reticulation vertex which is vertex 6. There are two displayed trees (shown in the center and on the right), which are each obtained by deleting one of the reticulation edges (1→6 or 5→6).

### Equivariant Phylogenetic Models

There are a number of classes of algebraic models that are useful to study, because they occur in phylogenetics practice or because they have nice mathematical properties, or both. Among the most useful classes to study for mathematical reasons in phylogenetics are the equivariant phylogenetic models. These are phylogenetic models where there is a certain group symmetry that is present among the states of the random variables. See (Casanellas and Fernández-Sánchez 2025) for more details on equivariant models. Specifically, let Σ denote the set of states of the random variables (e.g.  for DNA sequences, $\Sigma = \{\texttt {A}, \texttt {C}, \texttt {G}, \texttt {T} \}$). Let $\mathcal {G}$ be a group acting on Σ. A set of transition matrices *TM* is called equivariant relevant to $\mathcal {G}$ if for all M∈TM, all $x_1, x_2 \in \Sigma$ and all $g \in \mathcal {G}$

$$ M( x_2 | x_1) = M( g(x_2) | g(x_1)). $$

The model is a *generic equivariant model* relevant to the particular group action if it consists of all transition matrices that satisfy the given symmetry conditions. The *open generic equivariant model* consists of all transition matrices that satisfy the given equivariant condition but with no zeroes in the transition matrices. Adding the open condition can be useful if we want to impose the condition that a network is never allowed to have zero branch lengths (which correspond to the transition matrix being the identity matrix).

Examples of equivariant models include the Cavendar-Farris-Neyman model (CFN), the Jukes-Cantor model (JC), the Kimura 2 and 3 parameter models (K2P, K3P), the strand symmetric model (SSM), and the general Markov model (GMM). In general, we use, *TM*, to denote a set of transition matrices that define our model. Once *TM* is specified, the displayed tree model associated to a particular DAG *G* consists of all probability distributions that can arise on the observed variables, as the transition matrices range over all possible values in *TM*.

#### Example 3.4

Let $\Sigma = [k] = \{1, \ldots , k \}$, and let $\mathcal {G} = \langle 1 \rangle$ be the trivial group. Then the corresponding equivariant model is the general Markov model, consisting of all k×k transition matrices. Note that this set of transition matrices has k(k-1) free parameters, since there are no restrictions on it besides being a transition matrix.

#### Example 3.5

Let $\Sigma = \{\texttt {A}, \texttt {C}, \texttt {G}, \texttt {T} \}$, and let $\mathcal {G}$ be the symmetric group $S_4$ acting by permuting this set of size four. The corresponding general equivariant model is the Jukes-Cantor model consisting of all transition matrices of the form

$$ \begin{pmatrix} 1-3a & a & a & a \\ a & 1-3a & a & a \\ a & a & 1-3a & a \\ a & a & a & 1-3a \end{pmatrix}. $$

### Closure Properties of Sets of Transition Matrices

In this subsection, we consider ways that the set *TM* of transition matrices could be closed under various operations.

#### Definition 3.6

Let *TM* be a set of transition matrices. The set *TM* is *closed under multiplication* if for all $M^1, M^2 \in TM$, $M^1 M^2 \in TM$. The set *TM* is *closed under convex combinations* if for all $M^1, M^2 \in TM$ and $\delta \in [0,1]$, $\delta M^1 + (1- \delta ) M^2 \in TM$.

A more complicated property, that we will need for a result about 2-blobs, concerns convex combinations of marginal distributions.

#### Definition 3.7

Let *TM* be a set of transition matrices and *RD* be a set of corresponding root distributions in the transition model. Consider the set of two variable marginal distributions

$$ MD = \{ (M(x_b|x_a) \pi (x_a))_{x_a, x_b \in \Sigma } : M \in TM, \pi \in RD \}. $$

Then *TM* is *closed under marginal convex combinations*, if for all $\delta \in [0,1]$ and all $P^1, P^2 \in MD$, $\delta P^1 + (1-\delta )P^2 \in MD$.

It is straightforward to see that general equivariant models (and general open equivariant models) satisfy all three of these properties.

#### Proposition 3.8

Let *TM* be a set of transition matrices from a general equivariant phylogenetic model. Then *TM* is closed under multiplication, convex combinations, and marginal convex combinations. This is also true if *TM* is the open general equivariant phylogenetic model.

#### Proof

Being closed under multiplication of matrices follows immediately from the equivariant property. This is because the equivariant property says that the matrices are invariant under conjugation by certain permutation matrices, which holds after multiplying the matrices together.

Being closed under both types of convex combinations follows because the models are the intersection of a linear space with a convex set. □

The closure properties can be more subtle in the case where we are working with a set of transition matrices that is not as nice as a general equivariant model. For instance, if we take the set of transition matrices

$$ TM = \{ \exp (Qt) : Q \in RM, t \in [0, \infty ) \} $$

where *RM* is some collection of rate matrices. Such a set is generally not convex, and need not be closed under matrix multiplication. One model of this type that does satisfy all three properties is the *continuous time* Jukes-Cantor model. Let

$$ Q^{JC} = \begin{pmatrix} -3 & 1 & 1 & 1 \\ 1 & -3 & 1 & 1 \\ 1 & 1 & -3 & 1 \\ 1 & 1 & 1 & -3 \end{pmatrix}. $$

#### Proposition 3.9

Let $TM = \{ \exp (Q^{JC}t): t \in [0, \infty ) \}$ or $TM = \{ \exp (Q^{JC}t): t \in (0, \infty ) \}.$ Then *TM* is closed under multiplication, convex combinations, and marginal convex combinations.

#### Proof

Clearly

$$ \exp (Q^{JC}t_1)\exp (Q^{JC}t_2) = \exp (Q^{JC}(t_1 +t_2)) $$

so *TM* is closed under matrix multiplications.

Both *TM* and *MD* in this case are convex sets with

$$ TM = \left\{ \begin{pmatrix} 1-3a & a & a & a \\ a & 1-3a & a & a \\ a & a & 1-3a & a \\ a & a & a & 1-3a \end{pmatrix}: a \in [0, 1/4) \right\} $$

and

$$ MD = \left\{ \frac{1}{4} \begin{pmatrix} 1-3a & a & a & a \\ a & 1-3a & a & a \\ a & a & 1-3a & a \\ a & a & a & 1-3a \end{pmatrix} : a \in [0, 1/4) \right\} $$

(and for the case where we leave out t=0, remove the identity matrix). So the models will be closed under convex combinations and marginal convex combinations. □

### Splittability

Besides being closed under products of transition matrices and convex combinations, we will also be interested in sets of transition matrices that have another useful property we call splittability. Splittability is a feature of a set of transition matrices that will allow us to prove, in some instances, that two networks produce the same probability distributions. In a sense, splittability is like the inverse property to saying the family of distributions is closed under products.

#### Definition 3.10

Let *TM* be a set of transition matrices. The set *TM* is called *splittable* if for any *k* and any $M^1, \ldots , M^k \in TM$, there is a transition matrix N∈TM such that $M^1N^{-1}, \ldots , M^k N^{-1} \in TM$.

Clearly if *TM* contains the identity matrix, then it will be splittable, since we can take *N* to be the identity matrix.

#### Proposition 3.11

If *TM* is a general equivariant phylogenetic model, then *TM* is splittable.

#### Proof

The general equivariant phylogenetic model contains the identity matrix, so in the definition of splittable we can always take *N* to be the identity matrix. □

However, we are sometimes interested in restricting to sets of transition matrices that exclude the identity (since this corresponds to branch length zero), or excludes transition matrices which have zeros in them (as in the open general equivariant models, described above). Even in this case, there are often situations where the set of transition matrices is still splittable.

#### Proposition 3.12

Let *TM* be the set of transition matrices of a general open equivariant phylogenetic model (that is all entries of the transition matrices are positive). Then *TM* is splittable.

#### Proof

Let $M^1, \ldots , M^k \in TM$. Let $\Vert \cdot \Vert$ be a submultiplicative matrix norm (that is, $\Vert A B \Vert \le \Vert A \Vert \Vert B \Vert$). Let $B_\delta (M) = \{A: \Vert M - A \Vert < \delta \}$ be the open ball of radius δ centered at *M* with respect to the matrix norm and confined to the linear space of matrices with the given equivariant structure. Choose ϵ>0 smaller than

$$ \min _{i = 1}^k \sup \{ \delta : B_\delta (M^i) \subseteq TM \} . $$

By the conditions on our matrices, ϵ is strictly positive. Let

$$ \mu = \max ( 1, \max _{i = 1}^k \Vert M^i \Vert ). $$

Let *N* be any matrix in *TM* such that

$$ \Vert I - N \Vert < \frac{\epsilon }{2 \mu } . $$

Note that if A=I-N, then

$$ N^{-1} = \sum _{n = 0}^\infty A^n $$

so that

$$ \Vert N^{-1} \Vert \le \frac{1}{1 - \Vert A \Vert } \le 2 . $$

since we assume that $\Vert A \Vert$ is very small. Then

$$ \Vert I - N^{-1} \Vert = \Vert N^{-1} ( N - I) \Vert \le \Vert N^{-1} \Vert \Vert N- I \Vert \le \frac{\epsilon }{\mu }. $$

Then for each *i* we have that

$$ \Vert M^i - M^iN^{-1}\Vert = \Vert M^i (I - N^{-1}) \Vert \le \Vert M^i\Vert \Vert I - N^{-1} \Vert < \mu \cdot \frac{\epsilon }{\mu } = \epsilon . $$

So $M^i N^{-1}$ is a matrix of the correct equivariant form, and it has distance <ϵ to $M^i$. By our assumption on ϵ, $B_\epsilon (M^i) \subseteq TM$ so all the entries of $M^i N^{-1}$ are positive, and $M^i N^{-1} \in TM$ as desired. This shows that *TM* is splittable. □

## Local Modifications to DAGs

In this section, we want to discuss a useful property for looking at local features in a graphical model, and using that to compare if two graphical models describe the same family of probability distributions by looking at local features of the graph (4).

### Definition 4.1

Let G=(V,D) be a DAG. Let *A*, *B*, *C* be disjoint subsets of *V* with the following properties:

- For each vertex b∈B, every edge i→b has $i \in A \cup B$
- For each vertex b∈B, every edge b→i has $i \in B \cup C$
- For each vertex c∈C, every edge i→c has $i \in A \cup B \cup C$.
- For each vertex c∈C, there is no directed path starting at *c* and ending at a vertex a∈A.

We say that the triple of sets of vertices (*A*, *B*, *C*) gives a *local structure* in *G*.

**Fig. 4:**
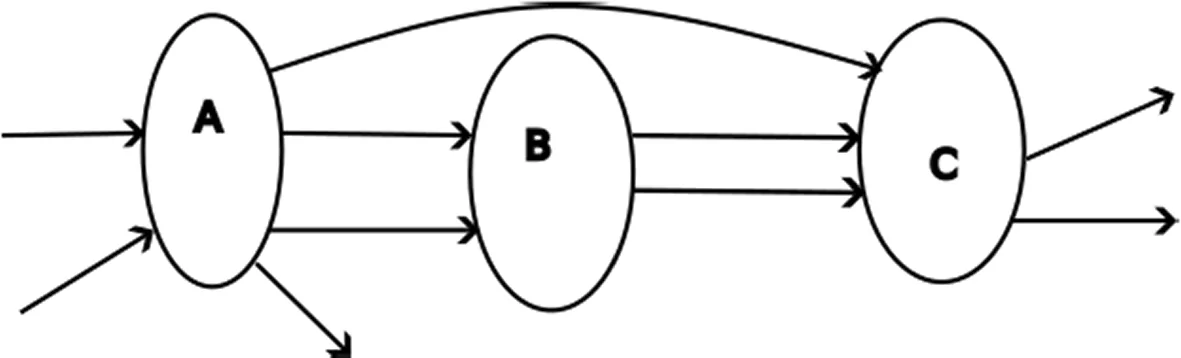
The diagram gives the idea of a local structure. Note that there can be directed edges within each of the groups *A*, *B*, and *C*

With these conditions on our graph, and the subsets *A*, *B*, *C*, we can directly calculate the conditional probability of $X_C$ given $X_A$ in the DAG *G* by the following formula:

$$ p_{C|A}(x_C | x_A) = \sum _{x_B \in \mathcal {R}_B} \prod _{i \in B \cup C } p_{i|\textrm{pa}(i)} ( x_i | x_{\textrm{pa}(i)} ). $$

To prove this formula, it suffices to prove that

$$ p_{B \cup C|A}(x_B, x_C | x_A) = \prod _{i \in B \cup C } p_{i|\textrm{pa}(i)} ( x_i | x_{\textrm{pa}(i)} ) $$

since the previous formula is obtained by marginalization of the second formula.

Let *S* be the ancestral set of $A \cup B \cup C$, that is, *S* consists of all vertices s∈V such that there is a path from *s* to some $t \in A \cup B \cup C$. A basic fact about sets *S* that are ancestral is that the joint distribution for probabilities in *S* has the form

$$ p_S(x_S) = \prod _{i \in S} p_{i|\textrm{pa}(i)}(x_i| x_{\textrm{pa}(i)}) $$

which holds because for each i∈S each of the parent sets $$\textrm{pa}(i) \subseteq S$$ when *S* is an ancestral set. Note that if (*A*, *B*, *C*) is a local structure for *G*, then the ancestral set $S^{\prime }$ for *A*, is simply $$S' = S \setminus (B \cup C).$$ Thus we have

$$ p_{S'}(x_{S'}) = \prod _{i \in S'} p_{i | \textrm{pa}(i)} (x_i | x_{\textrm{pa}(i)}). $$

From this we can see that

$$\begin{aligned} p_{B \cup C | S'}( x_B, x_C| x_{S'})= &  \frac{\prod _{i \in S} p_{i|\textrm{pa}(i)}(x_i| x_{\textrm{pa}(i)})}{\prod _{i \in S'} p_{i | \textrm{pa}(i)} (x_i | x_{\textrm{pa}(i)} ) }\\= &  \prod _{i \in B \cup C } p_{i|\textrm{pa}(i)} ( x_i | x_{\textrm{pa}(i)} ). \end{aligned}$$

However, since for each $i \in B \cup C$, our assumptions of being a local structure imply that each set $\textrm{pa}(i) \subseteq A \cup B$. In particular, this shows that the final formula only depends on elements in $A \cup B \cup C$, so that

$$ p_{B \cup C | S'}( x_B, x_C| x_{S'}) = p_{B \cup C | A}( x_B, x_C| x_{A}). $$

The fact that the conditional distribution for a local structure is local only to the variables *A*, *B*, *C* means that we can compare two graphical models that only differ by a change in a local structure. We call this a local modification.

### Definition 4.2

Let *G* be a DAG with a local structure (*A*, *B*, *C*). Let $$V' = V \setminus (A \cup B \cup C)$$ Let $G^{\prime }$ be a new DAG with vertex set $$V' \cup A \cup B' \cup C$$ that satisfies the following properties

- $\left(A,B^{\prime },C\right)$ is a local structure in $G^{\prime }$.
- Let $i,j \in V' \cup A$. Then $i \rightarrow j \in G$ if and only if $i \rightarrow j \in G'$.
- Let i∈C and $j\in V^{\prime }$. Then $$i \rightarrow j \in G$$ if and only if $$i \rightarrow j \in G'$$.

The graphs *G* and $G^{\prime }$ are called *local modifications* of each other.

In summary, graphs *G* and $G^{\prime }$ are local modifications of each other if they are exactly the same graph outside of the local structures (*A*, *B*, *C*) and $\left(A,B^{\prime },C\right)$. Note that in the "exactly the same" category we are requiring that any edge $$a \rightarrow a' \in G$$ also appears in $G^{\prime }$ .

### Example 4.3

The most basic example of a local modification is to subdivide an edge. Specifically, if a→c is any edge in *G*. We can take $$A = \{a\}, B = \emptyset , C = \{c\}$$ in *G*. In $G^{\prime }$ we replace the edge a→c with the pair of edges a→b and b→c where *b* is a new vertex and take $$B' = \{b\}$$. Then *G* and $G^{\prime }$ are local modifications of each other. This particular type of local modification is the subject of Proposition 4.5.

From the standpoint of comparing displayed tree models on different graphs, there are some situations where two graphs that are local modifications of each other can yield the same family of probability distributions. The structure of a local modification means that we can check this condition by purely looking at the induced structure of the conditional distributions in the two changed substructures.

Note that we use notation like $p_{G, A}(x_A)$ and $p_{G, A|C}(x_A | x_C)$, when we need to refer to the distributions that specifically come from the graph *G*.

### Theorem 4.4

Let G=(V,D) and $G^{\prime }=\left(V^{\prime },D^{\prime }\right)$ be two DAGS that are local modifications of each other with local structures (*A*, *B*, *C*) and $\left(A,B^{\prime },C\right)$ respectively. Suppose that the family of conditional distributions in the two models $$p_{G, C|A}(x_C|x_A)$$ and $$p_{G', C|A}(x_C|x_A)$$ are the same. Suppose further that each of the other families of distributions $$p_{i|\textrm{pa}(i)}(x_i, x_{\textrm{pa}(i)})$$ is the same in both models. Then the family of joint distributions with the variables in $$X_B$$ and $$X_{B'}$$ hidden variables are the same in both models.

### Proof

The distributions with *B* and $B^{\prime }$ hidden in both models looks like

$$\begin{aligned} p_{G}(x_{V \setminus B})= &  \sum _{x_B \in \mathcal {R}_B} \prod _{i \in V} p_{i| \textrm{pa}(i)}(x_i| x_{\textrm{pa}(i)}) \\= &  \prod _{i \in V \setminus (B \cup C)} p_{i| \textrm{pa}(i)}(x_i| x_{\textrm{pa}(i)} ) \sum _{x_B \in \mathcal {R}_B} \prod _{i \in B \cup C} p_{i| \textrm{pa}(i)}(x_i| x_{\textrm{pa}(i)} ) \end{aligned}$$

and

$$\begin{aligned} p_{G'}(x_{V' \setminus B'})= &  \sum _{x_{B'} \in \mathcal {R}_{B'}} \prod _{i \in V'} p_{i| \textrm{pa}(i)}(x_i| x_{\textrm{pa}(i)}) \\= &  \prod _{i \in V' \setminus (B' \cup C)} p_{i| \textrm{pa}(i)}(x_i| x_{\textrm{pa}(i)} ) \sum _{x_{B'} \in \mathcal {R}_{B'}} \prod _{i \in B' \cup C} p_{i| \textrm{pa}(i)}(x_i| x_{\textrm{pa}(i)} ). \end{aligned}$$

These factorizations are valid from (*A*, *B*, *C*) and $\left(A,B^{\prime },C\right)$ being local structures in *G* and $G^{\prime }$ respectively. In particular, every edge incident to *B* or $B^{\prime }$ only has edges from *A*, *C*, or $B,B^{\prime }$. And edges incoming to *A* do not involved *B*. These observations together allow to factor out all the terms $$p_{i, \textrm{pa}(i)}(x_i| x_{\textrm{pa}(i)})$$ where $$i \in V \setminus (B \cup C)$$ or $$i \in V' \setminus ( B' \cup C)$$.

By the assumptions of being a local modification, we have that $V \setminus (B \cup C) = V' \setminus (B' \cup C)$, and the edge structure of edges with heads in $V \setminus (B \cup C) = V' \setminus (B' \cup C)$ is exactly the same. Thus we have

$$ \prod _{i \in V \setminus (B \cup C)} p_{i| \textrm{pa}(i)}(x_i| x_{\textrm{pa}(i)} ) = \prod _{i \in V' \setminus (B' \cup C)} p_{i| \textrm{pa}(i)}(x_i| x_{\textrm{pa}(i)} ). $$

On the other hand, we know from above that

$$ p_{G, C|A}(x_C| x_A) = \sum _{x_B \in \mathcal {R}_B} \prod _{i \in B \cup C} p_{i| \textrm{pa}(i)}(x_i| x_{\textrm{pa}(i)} ) $$

and

$$ p_{G', C|A}(x_C| x_A) = \sum _{x_{B'} \in \mathcal {R}_{B'}} \prod _{i \in B' \cup C} p_{i| \textrm{pa}(i)}(x_i| x_{\textrm{pa}(i)} ). $$

Since we have assumed that the two models for the two local structures produce exactly the same family of conditional probability distributions $p_{G, C|A}(x_C| x_A)$ and $p_{G', C|A}(x_C| x_A)$, this means that the entire graphs $G_1$ and $G_2$ produce exactly the same probability distributions when $X_B$ are hidden variables. □

Here we provide one common example of how local modifications work.

### Proposition 4.5

Consider the displayed tree model on the DAG G=(V,D). Let a→c be an edge. Let $G^{\prime }=\left(V^{\prime },D^{\prime }\right)$ be the DAG obtained from *G* by replacing a→c with the path $$a \rightarrow b \rightarrow c$$. If the transition model *TM* is multiplicatively closed and splittable on transition matrices, and *b* is a hidden variable, then these two DAGs produce the same family of probability distributions on the observed variables.

### Proof

First we show that *G* and $G^{\prime }$ are local modifications of each other. Then we show that under the phylogenetic model the appropriate families of conditional distributions are the same, applying Theorem 4.4.

To see that *G* and $G^{\prime }$ are local modifications, first we have to take appropriate local structures. In *G* we take $$A = \textrm{pa}(c)$$, B=∅, and $$C = \{c\}$$. In $G^{\prime }$ we have the same *A* and *C*, and $$B = \{b\}$$. It is easy to check that these are both local structures. Indeed, in *G* nearly all conditions are vacuous since B=∅, and all edges i→c have i∈A by construction. Similarly, in $G^{\prime }$, we have any edge i→b is of the form a→b, any edge b→i has i=c, and any edge i→c has i=b or i∈A. This shows that *G* and $G^{\prime }$ are local modifications.

Now we must show that we get the same families of conditional distributions $$p_{c|A}(x_c| x_A)$$ in both *G* and $G^{\prime }$. Write $$A = \{a\} \cup A'$$. Then we have in *G*,

$$ p_{G, c|A}(x_c| x_A) = \pi ^c_a M^{ac}(x_c|x_a) + \sum _{a' \in A'} \pi ^c_{a'} M^{a'c}(x_c|x_{a'}) $$

whereas in $G^{\prime }$ we have

$$\begin{aligned} p_{G', c|A}(x_c| x_A)= &  \pi ^b_a \sum _{x_b} M^{ab}(x_b|x_a)M^{bc}(x_c|x_b) + \sum _{a' \in A'} \pi ^c_{a'} M^{a'c}(x_c|x_{a'}). \end{aligned}$$

We see that the distribution $$p_{G', c|A}(x_c| x_A)$$ from $G^{\prime }$ is a special case of the distribution $$p_{G, c|A}(x_c| x_A)$$ from *G* by taking $$\pi ^c_a = \pi ^b_a$$, and $$M^{ac} = M^{ab} M^{bc}$$, which is in the model since we assumed the class of transition matrices in multiplicatively closed.

On the other hand, with a distribution from *G*, we reverse this process since we assumed the model is splittable. Indeed, we take $\pi ^b_a = \pi ^c_a$ and by the splittable property of our model, there exists an *N* in the model such that we can take $M^{bc} = N$ and $M^{ab} = M^{ac}N^{-1}$. □

As a second example, we show that we can contract the edge from a reticulation vertex in some circumstances, without changing the set of probability distributions that arise.

### Proposition 4.6

Consider the displayed tree model on a DAG *G* which contains an edge b→c. Suppose that the outdegree of *b* is one and the indegree of *c* is one. Let $G^{\prime }$ be the graph obtained from *G* by contracting the edge b→c (that is, in $G^{\prime }$, we have an edge a→c for each edge a→b in *G*). If the transition model *TM* is multiplicatively closed and splittable, and *b* is a hidden variable in *G* then *G* and $G^{\prime }$ yield the same family of probability distributions.

### Proof

We need to show that *G* and $G^{\prime }$ are local modifications of each other, and that the corresponding conditional distributions families on those local modifications are the same.

In *G*, let $$A = \textrm{pa}(b)$$, $$B = \{b\}$$, and $$C = \{c\}$$. By construction (*A*, *B*, *C*) is a local structure in *G*. In $G^{\prime }$, we denote the vertex obtained by contracting the edge b→c by *c* as well, and take $B^{\prime }=\emptyset$. Note that we then have edges a→c for each a∈A. Again, $\left(A,B^{\prime },C\right)$ is a local structure in $G^{\prime }$ and the graphs are local modifications of each other.

Now we need to prove that the family of conditional distributions $p_{G, c|A}(x_c|x_A)$ and $p_{G', c|A}(x_c|x_A)$ in the two graphs are the same. In *G* we have

$$\begin{aligned} p_{G, c|A}(x_c | x_A)= &  \sum _{x_b =1}^{r_b} p_{b|A}(x_b| x_A) p_{c|b}(x_c|x_b) \\= &  \sum _{a \in A} \pi ^b_a \sum _{x_b=1}^{r_b} M^{ab}(x_b| x_a) M^{bc}(x_c|x_b) \end{aligned}$$

whereas in $G^{\prime }$ we have

$$\begin{aligned} p_{G', c|A}(x_c | x_A)= &  \sum _{a \in A} \pi ^c_a M^{ac}(x_c| x_a) \\ \end{aligned}$$

Hence, we see that each conditional distribution from *G* produces a distribution from $G^{\prime }$ by taking $$\pi ^c_a = \pi ^b_a$$ for all *a*, and $$M^{ac} = M^{ab} M^{bc}$$ for all *a*. This is valid because we assumed that the model is closed under multiplication of transition matrices.

On the other hand, with a distribution from $G^{\prime }$, we reverse this process since we assumed the model is splittable. Indeed, we take $$\pi ^b_a = \pi ^c_a$$ and by the splittable property of our model, there exists an *N* in the model such that we can take $$M^{bc} = N$$ and $$M^{ab} = M^{ac}N^{-1}$$ for each *a*. This shows that we can get exactly the same set of conditional distributions from each graph, and hence the two graphs give the same probability distributions by Theorem 4.4. □

**Fig. 5:**
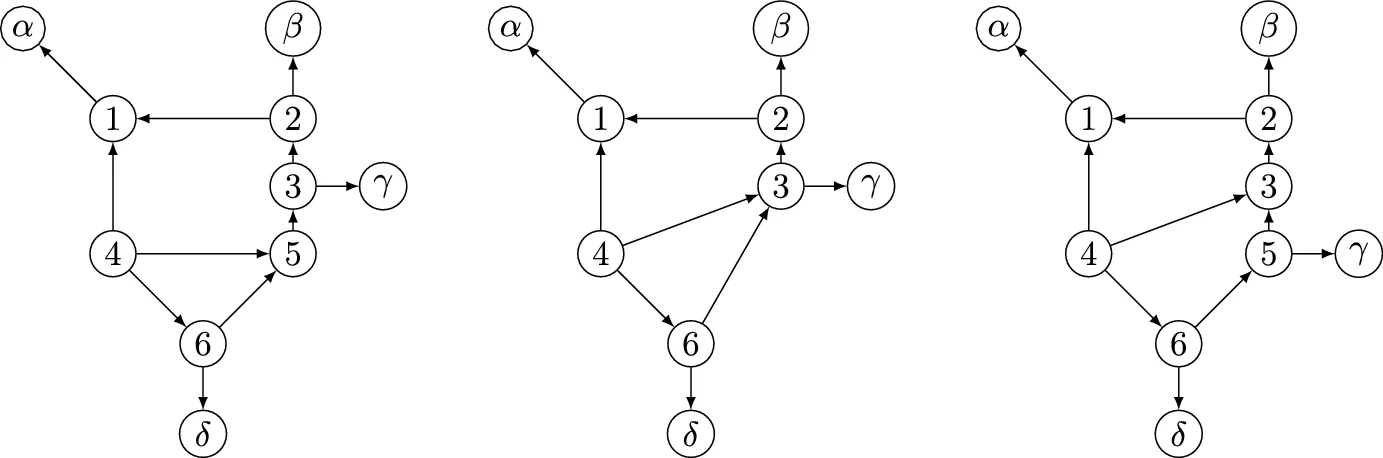
Example of an unusual containment between displayed tree models

### Example 4.7

Consider the three networks pictured in Figure 5, where we assume that only the leaf vertices are observed. In the network on the left, the edge 5→3 satisfies the conditions of Proposition 4.6. So if the transition model is multiplicatively closed and splittable, the network in the middle yields the same family of probability distributions as the network on the left.

For the network on the right, note that the edge 5→3 does not satisfy the conditions of Proposition 4.6. However, if our model of transition matrices contains the identity matrix, and we set this on that edge, then with that restriction, the edge does contract to produce the middle graph. Thus, if our transition model is multiplicatively closed, splittable, and contains the identity matrix, the network model on the right contains all the distributions from the model on the left. If we do not have the identity matrix as a transition matrix, but have the identity matrix as a limit, then at this point, we can say that distributions in the model on the left appears as limits of distributions of the right.

In the case of the Jukes-Cantor model, we checked the dimensions of the families of distributions from the left network and the right network and found that the dimension is 9 for the right network and 8 for the left network, so it is not possible that the two networks produce exactly the same family of distributions in that model. It is unclear what happens for other transition matrix structures.

## Stacked Reticulations

In this section, we explore an extension of Propositions 4.5 and 4.6 which is concerned with stacked reticulations. A stacked reticulation is a pair of vertices *b*, *c* such that *b* and *c* are each reticulations (that is, both have indegree greater than one), and such that b→c is an edge in the network. We show that under fairly broad conditions, it is possible to contract the edge b→c without changing the distributions that can arise from the displayed tree model. This result leads to a number of surprising cases where two binary networks can yield the same probability distributions, and hence lead to non-identifiability of the network models. These results suggest that stacked reticulations should likely be avoided in modeling phylogenetic networks under the displayed tree model.

### Theorem 5.1

Consider the displayed tree model on a DAG *G* which contains an edge b→c. Suppose that *b* has outdegree 1. Let $G^{\prime }$ be the graph obtained from *G* by contracting the edge b→c (that is, we have an edge a→c in $G^{\prime }$ for each edge a→b in *G*, and we keep the graph simple). If the transition model *TM* is multiplicatively closed, closed under convex combinations, and splittable, then the two graphs produce the same family of distributions when *b* is a hidden variable in *G*. The statement also holds if we require all reticulation probabilities to be positive.

### Proof

First, we show that the graphs *G* and $G^{\prime }$ are local modifications of each other. Then we show that the corresponding conditional distribution families are the same.

For the graph *G*, take $$B = \{b\}$$, $$C = \{c\}$$ and $$A = (\textrm{pa}(b) \cup \textrm{pa}(c)) \setminus \{b\}$$. Since we assumed there are no outgoing edges of *b* besides b→c, this shows that the triple (*A*, *B*, *C*) is a local structure in *G*. In the graph $G^{\prime }$ we take $B^{\prime }=\emptyset$. In $G^{\prime }$ we just have the edges a→c for all a∈A. So $\left(A,B^{\prime },C\right)$ is a local structure on $G^{\prime }$, and *G* and $G^{\prime }$ are local modifications of each other.

Now we analyze the conditional distributions of $$p_{G, c|A}$$ and $$p_{G', c|A}$$ in the two graphs. In *G* we use *M* to denote the transition matrices, and δ to denote reticulation parameters. In $G^{\prime }$, we use *N* and ϵ respectively. To examine $$p_{G, c|A}$$ and $$p_{G', c|A}$$, we first split the set *A* into three sets (based on *G*): $$A_1$$ is the set of vertices in *A* have *b* as a child but not *c*, $$A_2$$ is the set of vertices in *A* that have *c* as a child but not *b*. and $$A_3$$ is the set of vertices in *A* that have both *b* and *c* as children. With this division of *A*, we have the following form for $$p_{G, c|A}$$:

3 $$\begin{aligned} p_{G, c|A}(x_c|x_A)= &  \quad \sum _{a \in A_1} \delta ^b_a \delta ^c_b \sum _{x_b} M^{ab}(x_b|x_a) M^{bc}(x_c| x_b) \\ &  + \sum _{a \in A_2} \delta ^{c}_a M^{ac}(x_c|x_a) \nonumber \\ &  + \sum _{a \in A_3} \left( \delta ^b_a \delta ^c_b \sum _{x_b} M^{ab}(x_b|x_a) M^{bc}(x_c| x_b) + \delta ^{c}_a M^{ac}(x_c|x_a) \nonumber \right) . \end{aligned}$$

On the other hand, in $G^{\prime }$ we have

$$\begin{aligned} p_{G', c|A}(x_c| x_A)= &  \sum _{a \in A} \epsilon ^c_a N^{ac}(x_c | x_a). \end{aligned}$$

It is straightforward to see how to use the parameters from *G* to produce parameters in $G^{\prime }$. Indeed, for $$a \in A_1$$, take

$$ \epsilon ^c_a = \delta ^b_a \delta ^c_b, \quad N^{ac} = M^{ab}M^{bc}. $$

For $$a \in A_2$$ take

$$ \epsilon ^c_a = \delta ^c_a \quad N^{ac} = M^{ac}. $$

And for $a \in A_3$ take

$$ \epsilon ^c_a = \delta ^b_a \delta ^c_b + \delta ^c_a \quad N^{ac} = \frac{\delta ^b_a \delta ^c_b}{\delta ^b_a \delta ^c_b + \delta ^c_a} M^{ab}M^{bc} + \frac{\delta ^c_a}{\delta ^b_a \delta ^c_b + \delta ^c_a} M^{ac} $$

Since the substitution model *TM* is closed under matrix multiplication and convex combinations, this produces reticulation probabilities and transition matrices that belong to the conditional distribution for *c* given *A* in $G^{\prime }$ and if all the δ’s are positive so are the epsilons.

Now we need to show that any conditional distribution $$p_{G', c|A}$$ can be obtained from parameters from *G*. Note that the parametrization from *G* has many more free parameters than $G^{\prime }$, so there might be many ways to do this.

First of all, for $a \in A_2$, we take

$$ \delta ^c_a = \epsilon ^c_a \quad M^{ac} = N^{ac}. $$

Let $M^{bc}$ be a transition matrix in our model so that for each $a \in A_1 \cup A_3$

$$ M^{ab} = N^{ac}(M^{bc})^{-1} $$

is a transition matrix in the model, for each *a*. The matrix $M^{bc}$ exists because the model is splittable. For $a \in A_3$ take

$$ M^{ac} = N^{ac}. $$

We set

$$ \delta ^{c}_b = \sum _{a \in A_1} \epsilon ^c_a + \frac{1}{2} \sum _{a \in A_3} \epsilon ^c_a . $$

For $a \in A_1$, we take

$$ \delta ^b_a = \frac{\epsilon ^c_a}{\delta ^{c}_b}. $$

For $a \in A_3$, we take

$$ \delta ^b_a = \frac{\epsilon ^c_a}{2 \delta ^{c}_b} \quad \quad \delta ^c_a = \frac{1}{2} \epsilon ^c_a. $$

It is straightforward to see that these choices for the parameters in *G* will yield the parameters in $G^{\prime }$ when plugged into the formulas of Equation 3. The most complicated to check are for $$a \in A_3$$, but we see that

$$ \frac{\delta ^b_a \delta ^c_b}{\delta ^b_a \delta ^c_b + \delta ^c_a} M^{ab}M^{bc} + \frac{\delta ^c_a}{\delta ^b_a \delta ^c_b + \delta ^c_a} M^{ac} = \frac{1}{2} N^{ac} + \frac{1}{2} N^{ac} = N^{ac} $$

$$ \delta ^b_a \delta ^c_b + \delta ^c_a = \frac{1}{2} \epsilon ^{c}_{a} + \frac{1}{2} \epsilon ^{c}_{a} = \epsilon ^{c}_{a} $$

as desired. Furthermore, if all of ϵ’s were positive, then the constructed δ’s will be also. □

### Remark

Note that in terms of the conditions on the network, Theorem 5.1 appears to be a more general result than Propositions 4.5 and 4.6. However, Theorem 5.1 requires a more restrictive assumption on the transition model *TM* (specifically, it requires *TM* to be closed under convex combinations). So Propositions 4.5 and 4.6 are not a special case of Theorem 5.1.

**Fig. 6:**
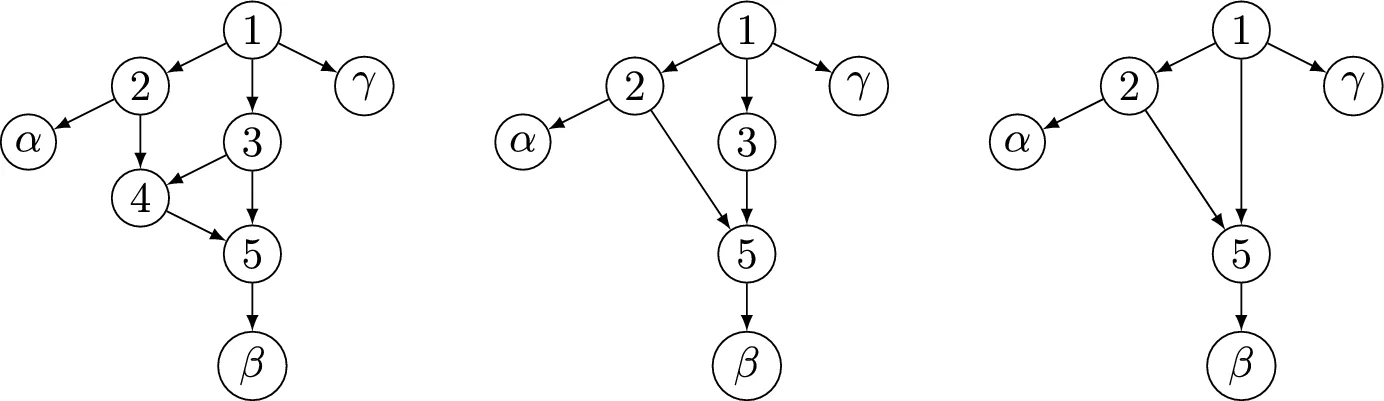
A stacked reticulation contraction of the edge 4→5. All three networks produce the same probability distributions on $\alpha , \beta , \gamma$

### Example 5.2

Consider the graphs in Figure 6. The network on the left has a stacked reticulation, and the local structure with $A = \{2,3\}, B = \{4\}, C = \{5\}$, and satisfies the conditions of Theorem 5.1. Hence, this graph yields the same probability distributions on the observed leaves $\alpha , \beta , \gamma$ as the network in the middle. Proposition 4.5 means that the network in the middle yields the same probability distributions as the network on the right. So this is a situation where a level-2 binary network will produce exactly the same family of probability distributions as a level-1 binary network.

### Example 5.3

Consider the three networks in Figure 1. Comparing the networks on the outside with the networks in the middle, we see that we can apply Theorem 5.1 to see that they yield the same families of probability distributions on observed leaves $\alpha , \beta , \gamma , \delta$. In particular, in both networks the stacked reticulation is on the edge 3→5. Contracting that edge in both cases yields the network in the middle.

One application of the stacked reticulation results concerns the presence of 2-blobs inside of a network, and the fact that these cannot be identified under the displayed trees model. Non-identifiability of 2-cycles in the displayed tree model is a common observation when studying the equivariant models (Gross and Long 2018; Gross et al. 2021), and Propositions 5.4 and 5.6 are a generalization of those results for arbitrary 2-blobs.

**Fig. 7:**
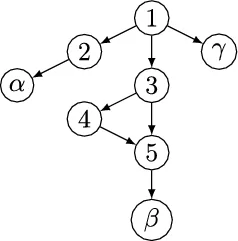
A network with a 2-blob consisting of vertices $\{3,4,5\}$

Recall that a *blob* in a network is a maximal 2-connected subgraph (that is, a subgraph that cannot be disconnected by deleting an edge). A 2-blob is a blob in a network that has two edges that connect it to other parts of the network. An example of a 2-blob appears in Figure 7, with the 2-blob itself being the subgraph consisting on vertices $\{3,4,5 \}$.

We distinguish two types of 2-blobs, depending on whether the root is part of the 2-blob, or not. If the root is part of the 2-blob we call it a *root blob*, otherwise it is a *non-root blob*. If a 2-blob is a root blob, there are two vertices $b_1$ and $b_2$ that are sinks of the blob, with edges $b_1 \rightarrow c_1$ and $b_2 \rightarrow c_2$ out of the blob. If a 2-blob is a non-root blob, the blob has a source $b_1$ and a sink $b_2$, with an edge $a \rightarrow b_1$ and an edge $b_2 \rightarrow c$, into and out of the blob, respectively.

### Proposition 5.4

Let *G* be a DAG with a non-root 2-blob, and let *B* be the set of vertices in the 2-blob. Let $$a \rightarrow b_1$$ be the edge pointing into the 2-blob and $$b_2 \rightarrow c$$ be the edge pointing out of the 2-blob. Suppose that all the random variables $$X_b$$ with b∈B are hidden variables. Let $G^{\prime }$ be the graph obtained from *G* by deleting all the vertices in *B* and incident edges and adding the edge a→c. If the transition model *TM* is multiplicatively closed, closed under convex combinations, and splittable, then the two graphs *G* and $G^{\prime }$ produce the same probability distributions.

### Proof

We use Theorem 5.1 repeatedly. First of all, because we have assumed that *TM* is closed under convex combinations, we can assume that the DAG *G* has no parallel edges/2-cycles. Indeed, if there were *k* parallel edges e→f, in the displayed tree model, the conditions distribution of $X_f| X_e$ would be

$$ \sum _{i = 1}^k\pi ^{f,i}_e M^{ef,i} $$

where the *i* in the superscripts is the indicator for the *i*th of the *k* copies of the edge e→f.

We can assume that all degree 2 vertices within the 2-blob have been suppressed by using Proposition 4.5. Then, since a 2-blob *B* does not consist only of the single edge $$b_1 \rightarrow b_2$$, then $$b_2$$ must have 2 or more parent vertices. None of those vertices can have a child that is outside of *B*. Since $$b_2$$ is a sink of *B*, there must be a vertex $$b_3$$ in *B* whose outdegree is 1, with an edge $$b_3 \rightarrow b_2$$. By Theorem 5.1, we can contract this edge without changing the family of distributions that can arise. This process can be repeated until we are left with only the edge $$b_1 \rightarrow b_2$$. Both of the degree two vertices $$b_1$$ and $$b_2$$ can be contracted using Proposition 4.5. The end result of this process is the graph $G^{\prime }$. □

In the case of a root 2-blob, we end up with a weaker statement, because not every root 2-blob can be eliminated using the removal of stacked reticulations. We also need the concept of closed under marginal convex combinations (Definition 3.7), and a common condition of a phylogenetic model we call *root unidentifiable*.

### Definition 5.5

A pair of transition matrices *TM* and root distributions *RD* is *root unidentifiable* if for any $M^1 \in TM$ and $\pi ^1 \in RD$, there are $M^2 \in TM$ and $\pi ^2 \in RD$ such that

$$ M^1(x_b| x_a) \pi ^1(x_a) = M^2(x_a | x_b) \pi ^2(x_b) $$

for all $$x_a, x_b \in \Sigma $$.

The name “root unidentifiable” is used because this condition means that any joint distribution from the model obtained from the graph 1→2, could also have been obtained from the graph 2→1, with a different choice of rate matrix and root distribution. This property occurs in many models (for example, all equivariant models satisfy it) and it implies that in a larger tree, the location of the root cannot be identified since it is possible to switch directions of the edges without changing the resulting probability distributions that arise.

### Proposition 5.6

Let *G* be a DAG with a root 2-blob, and let *B* be the set of vertices in the 2-blob. Let $$b_1$$ and $$b_2$$ be the two sinks of the blob, with outgoing edges $$b_1 \rightarrow c_1$$ and $$b_2 \rightarrow c_2$$. Suppose all random variables $$X_b$$ with b∈B are hidden variables. Let $G^{\prime }$ be the graph obtained from *G* by deleting all the vertices in *B* except $$b_1$$ and $$b_2$$, and adding a vertex *a* with edges $$a \rightarrow b_1$$ and $$a \rightarrow b_2$$, and having $$X_a$$ be a hidden variable. If the transition model *TM* is multiplicatively closed, closed under marginal convex combinations, and root unidentifiable, then every distribution from *G* can be realized as a distribution from $G^{\prime }$.

### Proof

It suffices to compute the marginal distribution of $(X_{b_1}, X_{b_2})$ in both graphs. To see this, let *C* be the set of all vertices below the 2-blob. Since the 2-blob is ancestral to all other vertices in *G*, so the marginal distribution on $C' = \{b_1, b_2\} \cup C$ is

$$\begin{aligned} p_{G,C }(x_{b_1}, x_{b_2}, x_C)= &  p_{G,\{b_1, b_2\} }(x_{b_1}, x_{b_2}) p_{G,C|\{b_1, b_2\} }(x_C | x_{b_1}, x_{b_2}) \\ p_{G',C }(x_{b_1}, x_{b_2}, x_C)= &  p_{G',\{b_1, b_2\} }(x_{b_1}, x_{b_2}) p_{G',C|\{b_1, b_2\} }(x_C | x_{b_1}, x_{b_2}). \end{aligned}$$

However,

$$ p_{G,C|\{b_1, b_2\} }(x_C | x_{b_1}, x_{b_2}) = p_{G',C|\{b_1, b_2\} }(x_C | x_{b_1}, x_{b_2}) $$

by assumption since that only involves edges that are not in the blob. Hence, we can suppose that *G* just consists of the 2-blob itself, and $G^{\prime }$ is the graph with only three vertices $$a, b_1, b_2$$, and edges $$a \rightarrow b_1$$, $$a \rightarrow b_2$$.

In the graph *G*, the marginal distribution is obtained by summing over all possible ways to choose reticulation edges at each reticulation vertex. The resulting probability distribution when each of the those reticulation edge choices are made is a 2-leaf tree. Those distributions for 2-leaf trees are weighted by the reticulation probabilities associated to each of those edge choices. Since the model is root unidentifiable and multiplicatively closed, each one of those distributions is a marginal distribution from the model on a two leaf tree, which belongs to the associated set of 2-variable marginal distributions, *MD*. Since the transition model is closed under marginal convex combinations, this shows that every distribution from *G* can be realized as a distribution from $G^{\prime }$. □

On the other hand, every distribution coming from the simplified graph $G^{\prime }$ arises as a limit of distributions from *G*. This gives a partial converse to Proposition 5.6.

### Proposition 5.7

Let *G* be a DAG with a root 2-blob, and let *B* be the set of vertices in the 2-blob. Let $$b_1$$ and $$b_2$$ be the two sinks of the blob, with outgoing edges $$b_1 \rightarrow c_1$$ and $$b_2 \rightarrow c_2$$. Suppose all random variables $$X_b$$ with b∈B are hidden variables. Let $G^{\prime }$ be the graph obtained from *G* by deleting all the vertices in *B* except $$b_1$$ and $$b_2$$, and adding a vertex *a* with edges $$a \rightarrow b_1$$ and $$a \rightarrow b_2$$, and having $$X_a$$ be a hidden variable.

Suppose that *TM* is splittable and contains transition matrices that are arbitrarily close to the identity matrix. Then any distribution produced by the network $G^{\prime }$ is in the limit of the set of distributions produced by the network *G*.

### Proof

As in the proof of Proposition 5.6, we can suppose that *G* just consists of the 2-blob itself, and $G^{\prime }$ is the graph with only three vertices $$a, b_1, b_2$$, and edges $$a \rightarrow b_1$$, $$a \rightarrow b_2$$. So a distribution *P* that is a marginal distribution associated to the graph $G^{\prime }$ is of the form

$$ P(x_{b_1}, x_{b_2}) = \sum _{x_a} \pi (x_a) M^{ab_1}(x_{b_1} | x_a) M^{ab_2}(x_{b_2} | x_a). $$

In *G*, let $p_1$ be any path from the root to $b_1$ and let $p_2$ be any path from the root to $b_2$. Let the vertices on $p_1$ be $a, c_1, \ldots , c_k, b_1$ and the vertices on the path $p_2$ be $a, d_1, \ldots , d_l, b_2.$

Suppose that $p_1$ and $p_2$ do not have any common vertices besides the root *a*. Since *TM* is splittable, we can choose a sequence of matrices, $M^{ac_1}, M^{c_1c_2}, \ldots , M^{c_kb_1} \in TM$ such that

$$ M^{ab_1} = M^{ac_1}M^{c_1c_2} \cdots M^{c_kb_1}. $$

Similarly, there is a sequence of matrices $M^{ad_1}, M^{d_1d_2}, \ldots , M^{d_lb_2} \in TM$ such that

$$ M^{ab_2} = M^{ad_1}M^{d_1d_2} \cdots M^{d_lb_2}. $$

For each of the edges in the paths $$p_1$$ and $$p_2$$ assign the corresponding transition matrix produced above. Make the root distribution π in *G* the same as the root distribution in $G^{\prime }$. For all the other edges in *G*, take arbitrary matrices in *TM*. For the reticulation parameters, for each reticulation that involves one of the edges on either the path $$p_1$$ or $$p_2$$, choose the probability 1-ϵ for the edge involved in the path, and distribute ϵ among the other reticulation edges (since there may be more than two edges entering a reticulation). For all other reticulations, choose the reticulation probabilities arbitrarily.

When ϵ=0, the marginal probability of $$(X_{b_1}, X_{b_2})$$ is completely determined by the probability on the pair of paths $$p_1$$ and $$p_2$$, which is equal to the distribution *P*. Hence, as $$\epsilon \rightarrow 0$$, the probabilities in the model for $G^{\prime }$ converge to the distribution *P*.

Finally, suppose that $p_1$ and $p_2$ share a vertex besides the root. In that case, we can treat the lowest shared such vertex as the root, and use the argument above. Indeed, in addition to the argument above, we use, for each edge not on the paths $p_1$ and $p_2$, a sequence of matrices $M_\epsilon$ converging to the identity matrix as $\epsilon \rightarrow 0$ and put that matrix $M_\epsilon$ on each edge not in $p_1$ and $p_2$. □

Note that in Proposition 5.7, we needed to take a limit, because it is typically assumed that reticulation probabilities should all be in (0, 1) on a network. Similarly, a more simple approach could also involve using identity matrices on most edges, but that might be excluded by our considerations on the transition model *TM*, e.g. if we want to assume all branch lengths are >0.

The combination of Propositions 5.6 and 5.7 is usually enough that one can ignore root 2-blobs in identifiability analysis of the displayed tree model. Indeed, if we consider a model that is a semialgebraic set for each network *G*, then if *G* and $G^{\prime }$ are related as above, and the transition model is multiplicatively closed, closed under marginal convex combinations, time reversible, and splittable, then the two models $$M_G$$ and $$M_{G'}$$ have the same topological closure, and $$M_G \subseteq M_{G'}$$. Hence, if *H* is another network and $$M_{G'}$$ and $$M_H$$ are distinguishable, then so are $$M_G$$ and $$M_{H}$$.

It remains an open problem to show that in the case of a root 2-blob, the two networks *G* and $G^{\prime }$ produce the exact same probability distributions, under a suitable restriction on the transition model *TM*.

## Non-Identifiability of Numerical Parameters through Conditional Distributions

In this section, we point out some loss of identifiability that can occur in the displayed tree model. In particular, it can happen that the conditional distribution at a reticulation node has lower dimension than the number of parameters that go into it, which results in a loss of identifiability in those numerical parameters.

To this end, consider the family of conditional distributions in the displayed tree model at a single reticulation node. Recall that a general conditional distribution at a reticulation node will have the form

$$ p_{c | \textrm{pa}(c)}(x_c | x_{\textrm{pa}(c)}) = \sum _{a \in \textrm{pa}(c)} \pi ^c_a M^{ac}(x_c|x_a) $$

which gives a restricted class of conditional distributions. In fact, we can see that if $m = \#\textrm{pa}(c)$ and we consider random variables with *k* states, then in the completely general DAG model, the dimension of the space of such conditional distributions is $(k-1)k^m$. This is because for each of the $k^m$ states of $x_{\textrm{pa}(c)}$ we get a probability distribution in $\Delta _k$, which has k-1 free parameters.

On the other hand, in terms of parametrizing the set of conditional distributions that arise from the network model, we see that there are m-1 parameters for the $\pi ^c_a$ parameters, and each transition matrix gives (k-1)k parameters, for a total of m(k-1)k+m-1 parameters. There are even fewer parameters for other equivariant models.

It turns out that the conditional distributions that arise from network model have to satisfy many linear relations. We will focus just on the case of the general Markov model for an arbitrary reticulation.

### Proposition 6.1

Consider a general reticulation vertex *c* with $\textrm{pa}(c) = A$, and edges a→c for a∈A as part of the displayed tree model. Then the conditional distribution $p_{c|A}(x_c|x_A)$ satisfies the relations

$$ p_{c|A}(x_c|x_A) + p_{c|A}(x_c|y_A) = p_{c|A}(x_c|x'_A) + p_{c|A}(x_c|y'_A) $$

for all $x_c \in \Sigma$, $x_A, y_A \in \Sigma ^A$, where $x'_A$ and $y'_A$ are any vectors of states such that for all a∈A $\{x_a, y_a \} = \{x'_a, y'_a \}$ as sets.

### Proof

For a distribution in the network model we have

$$\begin{aligned} p_{c|A}(x_c|x_A) + p_{c|A}(x_c|y_A)= &  \sum _{a \in A} \pi ^c_a M^{ac}(x_c|x_a) + \sum _{a \in A} \pi ^c_a M^{ac}(x_c|y_a)\\= &  \sum _{a \in A} \pi ^c_a(M^{ac}(x_c| x_a) + M^{ac}(x_c | y_a) ) \\= &  \sum _{a \in A} \pi ^c_a(M^{ac}(x_c| x'_a) + M^{ac}(x_c | y'_a) ) \\= &  \sum _{a \in A} \pi ^c_a M^{ac}(x_c|x'_a) + \sum _{a \in A} \pi ^c_a M^{ac}(x_c|y'_a)\\= &  p_{c|A}(x_c|x'_A) + p_{c|A}(x_c|y'_A). \end{aligned}$$

This follows because the condition that for all a∈A we have $\{x_a, y_a \} = \{x'_a, y'_a \}$ guarantees that the same terms appear in both sums for each *a*. □

The equations from Proposition 6.1 restrict the conditional distributions that come from the displayed tree model to a low dimensional space.

We now consider consequences for different phylogenetic modeling situations.

### Theorem 6.2

Consider the general Markov model on *k* states, and a reticulation node *c* with $\textrm{pa}(c) = A$. Let m=#A. Then the space of conditional distributions $p_{c|A}$ that can arise from this model has dimension (k-1)(m(k-1)+1). In particular, since there are there m(k-1)k+m-1 parameters that go into the displayed trees model to describe this conditional distribution, this results in a loss of (m-1)k dimensions.

### Proof

We can use Proposition 6.1 to calculate the number of linearly independent parameters in the linear space of conditional distributions $p_{c|A}$ that could come from the displayed tree model. Indeed, for each fixed value of $x_c$ consider the set of coordinates

$$ \{p(x_c| x_A): \#\{ a \in A : x_a \ne 1 \} \le 1 \} . $$

If we add any coordinate $p(x_c|x_A)$ with $x_A$ outside of this set, there will be a linear relation using the relations from Proposition 6.1.

On the other hand, we claim that the set of coordinates

$$ S = \{p(x_c| x_A): x_c \in [k-1] \text{ and } \#\{ a \in A : x_a \ne 1 \} \le 1 \} $$

is algebraically independent. Indeed, for each fixed $x_c$, there is no overlap of the entries of the matrices $M^{ac}$ that are involved (and we leave off $x_c = k$, since otherwise we would get a relation since $\sum _{x_c} p_{c|A}(x_c | x_A) = 1$). On the other hand, for each $x_A$, where $x_a \ne 1$ and each $x_c$, $p_{c|A}(x_c|x_A)$ is the unique place where the algebraically independent entry $M^{ac}(x_c|x_a)$ appears. Thus shows that *S* is algebraically independent.

Note that #S=(k-1)(m(k-1)+1). On the other hand, the number of free parameters in each matrix is k(k-1), there are *m* of them, and there are m-1 reticulation parameters for a total of mk(k-1)+m-1. But

mk(k-1)+m-1-((k-1)(m(k-1)+1))=(m-1)k

which gives the indicated loss of dimension. □

Generalizations of Theorem 6.2 for general equivariant models appear in Casanellas and Fernández-Sánchez (2025), including a useful reparametrization of the conditional distribution when the reticulation node only has two parents.

## Conditional Independence and the Ranks of Flattenings

In this section, we explore how conditional independence structures in graphical models can be used to deduce new results about rank conditions on flattening matrices in probability distributions that come from the displayed tree model. These rank of flattening results generalize the classic results for trees (Allman and Rhodes 2008). For level-1 networks, ranks for flattenings can be used as a tool to prove identifiability results in the displayed tree model (Casanellas and Fernández-Sánchez 2025).

First, we recall the definition of conditional independence of random vectors.

### Definition 7.1

Let *X* be a discrete random vector with index set *V* and let *A*, *B*, and *C* be disjoint subsets of *V*. We say that $X_A$ is conditionally independent of $X_B$ given $X_C$ (denoted $X_A \perp \hspace{-5.5pt}\perp X_B | X_C$) if for all $x_A \in \mathcal {R}_A$, $x_B\in \mathcal {R}_B$, and $x_C \in \mathcal {R}_C$ we have

$$ P(X_A = x_A, X_B = x_B | X_C = x_C) = P(X_A = x_A | X_C = x_C) P(X_B = x_B | X_C = x_C). $$

The key concept to describe conditional independence constraints in DAGs is the notion of *d*-separation.

### Definition 7.2

Let G=(V,D) be a DAG.

- A *chain* in a digraph is a sequence of vertices $\pi = v_1, v_2, \ldots , v_k$ such that for each *i* either $v_i \rightarrow v_{i+1}$ or $v_{i+1} \rightarrow v_i$ is in *D*.
- A *collider* is a sequence of vertices *a*, *b*, *c* such that a→b and c→b are edges.
- A chain π from *a* to *b* is said to be *blocked* by a set S⊆V if there is a vertex $v_i \in \pi$ such that either
  - $v_i \in S$ and $v_{i-1}, v_i, v_{i+1}$ is not a collider or
  - $v_i$ and all its descendants are not in *S* and $v_{i-1}, v_i, v_{i+1}$ is a collider.
- Two sets of vertices *A* and *B* are said to be *d*-separated given a set *C* if for all a∈A and b∈B, every chain from *a* to *b* is blocked by *C*.

### Proposition 7.3

A conditional independence statement $X_A \perp \hspace{-5.5pt}\perp X_B | X_C$ holds for all distributions associated to the DAG *G* if and only if *A* and *B* are *d*-separated by *C* in *G*.

See (Lauritzen 1996) Section 3.2.2 for more on d-separation and a proof of Proposition 7.3.

The conditional independence statement being true for a probability distribution means that certain rank conditions hold on flattened and marginalized versions of the joint probability distribution.

### Definition 7.4

Let *A*, *B*, *C* be disjoint subsets of *V*. Let *P* be a joint distribution. The *flattening matrix* $\textrm{Flat}(A,B)(P)$ is the matrix whose rows are indexed by $\mathcal {R}_A$ and columns are indexed by $\mathcal {R}_B$, and where the entry in the $(x_A, x_B)$ row and column is the marginal probability

$$ P(X_A = x_A, X_B = x_B). $$

The *conditional flattening matrix* is the matrix $\textrm{Flat}(A,B | C, x_C)(P)$ is the matrix whose rows are indexed by $\mathcal {R}_A$ and columns are indexed by $\mathcal {R}_B$, and where the entry in the $(x_A, x_B)$ row and column is the probability

$$ P(X_A = x_A, X_B = x_B, X_C = x_c). $$

### Example 7.5

Suppose that we have 5 random variables, $X_1, \ldots , X_5$ each of which is binary. Let $A = \{1,2\}, B = \{3,4\}, C = \{5\}$. Let

$$ p_{x_1x_2x_3x_4x_5} = P(X_1 = x_1, X_2 = x_2, X_3 = X_3, X_4 = x_4, X_5 = x_5). $$

Then

$$ \textrm{Flat}(12,34 | 5, x_5)(P) = \begin{pmatrix} p_{0000x_5} & p_{0001x_5} & p_{0010x_5} & p_{0011x_5} \\ p_{0100x_5} & p_{0101x_5} & p_{0110x_5} & p_{0111x_5} \\ p_{1000x_5} & p_{1001x_5} & p_{1010x_5} & p_{1011x_5} \\ p_{1100x_5} & p_{1101x_5} & p_{1110x_5} & p_{1111x_5} \\ \end{pmatrix} $$

### Proposition 7.6

Let *P* be a probability distribution that satisfies the conditional independence statement $X_A \perp \hspace{-5.5pt}\perp X_B | X_C$. Then for each $x_C \in \mathcal {R}_C$, the conditional flattening matrix $\textrm{Flat}(A,B | C, x_C)(P)$ has rank ≤1.

### Proof

This follows from the definition of conditional independence given above. Indeed, we have the equation

$$ P(X_A = x_A, X_B = x_B | X_C = x_C) = P(X_A = x_A | X_C = x_C) P(X_B = x_B | X_C = x_C). $$

which expresses the matrix $\textrm{Flat}(A,B | C, x_C)(P)/ P(X_C = x_C)$ as a rank one matrix provided $P(X_C)$ is not zero. So clearing denominators shows that $\textrm{Flat}(A,B | C, x_C)(P)$ is a rank one matrix (or the zero matrix if $P(X_C = x_C) = 0$). □

### Corollary 7.7

Let *P* be a probability distribution that satisfies the conditional independence statement $X_A \perp \hspace{-5.5pt}\perp X_B | X_C$. Then the flattening matrix $\textrm{Flat}(A,B)(P)$ has rank $\le \#\mathcal {R}_C$.

### Proof

We have that

$$ \textrm{Flat}(A,B)(P) = \sum _{x_C \in \mathcal {R}_C} \textrm{Flat}(A,B | C, x_C)(P) . $$

Since each $\textrm{Flat}(A,B | C, x_C)(P)$ has rank ≤1, and the rank is subadditive, we see that

$$\begin{aligned} {{\,\textrm{rank}\,}}\textrm{Flat}(A,B)(P) \le \#\mathcal {R}_C . \end{aligned}$$

□

These statements hold in arbitrary DAG models, without reference to the models arising from a network. In the network models, we can use this result to deduce rank conditions on flattening matrices when we cut edges in the network. This follows from a simple observation.

### Theorem 7.8

Let *G* be a DAG under the displayed tree model with an equivariant Markov model with *k* state random variables. Let *E* be a set of edges of *G* such that the removal of *E* from *G* disconnects the graph into (at least) 2 subgraphs. Let *A* and *B* be the two leaf sets of two disconnected components. Then

$$ {{\,\textrm{rank}\,}}\textrm{Flat}(A,B)(P) \le k^{\#E} $$

for any distribution *P* in the model.

### Proof

First, we perform a local modification to *G*. For each edge a→b in *E*, we replace it with a path $$a \rightarrow c \rightarrow b$$ (for some new independent vertex *c* for each edge). Let $G^{\prime }$ be the resulting graph. According to Proposition 4.5, these local modifications of *G* and $G^{\prime }$ yield the same family of probability distributions if all the intermediate *c* vertices are hidden. Let *C* be the collection of all of those *c* vertices that were added into $G^{\prime }$, and *A* and *B* the two sets of leaves that are disconnected by removing the edge set *E*.

Since *A* and *B* are disconnected by removing the edges *E* in *G*, this means that in $G^{\prime }$, every chain from an a∈A to a b∈B must pass through a vertex c∈C. However, since each c∈C, only appears on a path $$a' \rightarrow c \rightarrow b'$$ in $G^{\prime }$, this chain is blocked by *C*. This implies that $$X_A \perp \hspace{-5.5pt}\perp X_B | X_C$$ in $G^{\prime }$. By Corollary 7.7, $${{\,\textrm{rank}\,}}\textrm{Flat}(A,B)(P) \le k^{\#E}$$. □

### Example 7.9

Consider the 6-sunlet network in Figure 3. Removing the two edges 2→1 and 4→5 separates the leaves into two groups $A = \{\alpha , \zeta , \epsilon \}$ and $B = \{\beta , \gamma , \delta \}$. So the flattening matrix $\textrm{Flat}(A,B)(P)$ has

$$ {{\,\textrm{rank}\,}}\textrm{Flat}(A,B)(P) \le k^2 $$

for a *k* state phylogenetic model. Note that $\text {Flat}(A,B)(P)$ is a $k^3 \times k^3$ matrix in this case, so this rank condition is giving a nontrivial restriction on the matrix $\text {Flat}(A,B)(P)$.

As a complement to Theorem 7.8, results in the literature on ranks of flattenings of tensors associated to phylogenetic trees show in some situations that we can derive lower bounds on the rank of a flattening. To explain this we need the notion of the parsimony score of a split *A*|*B* which may not be displayed by a particular tree.

### Definition 7.10

Let *T* be a tree and let *A*|*B* be a split of the leaves. A labeling of the vertices of *T* with $\{0,1\}$ is compatible with the split *A*|*B* if every a∈A gets label 0 and every b∈B gets label 1. The parsimony score of *A*|*B* on *T* is the smallest number of 0/1 edges in any compatible labeling of *T*. Denote this by $\ell _T(A|B)$.

Note that the split *A*|*B* appears in the tree *T* if and only if $\ell _T(A|B) = 1$.

### Theorem 7.11

Let *G* be a DAG evolving under the displayed tree model for random variables with *k* states. Let *A*|*B* be a partition of the leaves of *G*. Suppose that one of the displayed trees *T* of *G* has parsimony score $\ell _T(A|B)$. Then for a generic probability distribution *P* in the model

$$ {{\,\textrm{rank}\,}}\textrm{Flat}(A,B)(P) \ge \min ( k^{\#A}, k^{\#B}, k^{\ell _T(A|B)} ). $$

### Proof

The main feature we use is that the rank of a matrix is upper-semicontinuous, which implies that if there is a value of the parameters that achieves a particular rank, then generic points will also have rank at least that value. The probability distributions for a fixed displayed tree *T* always arise in the closure of the displayed tree model (e.g. by setting all the reticulation parameters associated to the edges in that tree to 1, and all other reticulation parameters to zero). So it suffices to show that the distribution for a tree with the fixed parsimony score $\ell _T(A|B)$ gives the appropriate rank. However, this result for trees is show in (Casanellas and Fernández-Sánchez 2011, Prop. 3.1) and (Snyman et al. 2023, Thm. 8). □

### Example 7.12

Consider the 6-sunlet network from the left of Figure 3 and consider the split of the leaves $A|B = \{\alpha , \gamma , \epsilon \}|\{\beta , \delta , \zeta \}$. In both of the two displayed trees for this network, the parsimony score is $\ell _T(A|B) = 3$ (which is straightforward to see since the labeling alternates as it goes around the sunlet). So this shows that for a generic *P* from the network model

$$ {{\,\textrm{rank}\,}}\textrm{Flat}(A,B)(P) \ge k^3 $$

by Theorem 7.11. However, $\textrm{Flat}(A,B)(P)$ is a $k^3 \times k^3$ matrix, so the rank is generically equal to $k^3$.
